# Neural control and precision of flight muscle activation in *Drosophila*

**DOI:** 10.1007/s00359-016-1133-9

**Published:** 2016-12-09

**Authors:** Fritz-Olaf Lehmann, Jan Bartussek

**Affiliations:** Department of Animal Physiology, University of Rostock, Albert-Einstein-Str. 3, 18059 Rostock, Germany

**Keywords:** Insect flight, Wing kinematics, Muscle activation timing, Muscle power control, *Drosophila*

## Abstract

Precision of motor commands is highly relevant in a large context of various locomotor behaviors, including stabilization of body posture, heading control and directed escape responses. While posture stability and heading control in walking and swimming animals benefit from high friction via ground reaction forces and elevated viscosity of water, respectively, flying animals have to cope with comparatively little aerodynamic friction on body and wings. Although low frictional damping in flight is the key to the extraordinary aerial performance and agility of flying birds, bats and insects, it challenges these animals with extraordinary demands on sensory integration and motor precision. Our review focuses on the dynamic precision with which *Drosophila* activates its flight muscular system during maneuvering flight, considering relevant studies on neural and muscular mechanisms of thoracic propulsion. In particular, we tackle the precision with which flies adjust power output of asynchronous power muscles and synchronous flight control muscles by monitoring muscle calcium and spike timing within the stroke cycle. A substantial proportion of the review is engaged in the significance of visual and proprioceptive feedback loops for wing motion control including sensory integration at the cellular level. We highlight that sensory feedback is the basis for precise heading control and body stability in flies.

## Introduction

Precision of locomotor behavior is the key to the evolutionary success of animals and humans because motor control is often challenged in tasks with fastidious demands (Willigenburg et al. 2013). Successful handling of locomotor tasks, however, is highly prone to neuromuscular noise. Noise typically causes alterations in locomotor control and motor systems thus require elaborated sensory feedback for optimized performance (Roth et al. 2013). Receptor noise as well as random external perturbations from the environment may affect sensory precision though, changing muscle activation patterns and thus the resulting kinematics of the animal’s body appendages. In general, experimental and computational approaches have shown that noise in nervous systems greatly contribute to both cellular and behavioral variability (Faisal et al. 2008). In humans and monkeys, elevated motor precision is relevant in a large context of various motor behaviors, including goal-directed tasks such as control of precision grip by fingers and thumbs while lifting weights and grabbing objects with rough or slippery surfaces (e.g., Johansson and Westling 1984, 1988; Takei and Seki 2013), and equilibrium reflexes during the cortical control of normal gait, precision stepping (e.g., Fuglevand 2011; Koenraadt et al. 2013), and precision control of trunk movement (Willigenburg et al. 2013). While goal-directed tasks require neural forward models which modify inner-loop feedback control systems, equilibrium reflexes are typically controlled by means of negative feedback loops.

In most vertebrates, muscular precision typically depends on the activity of a synergistic ensemble of numerous motor units, controlling mechanical forces and their dynamic changes via an elevated number of independently working muscle fibers and motor neurons (Thelen and Anderson 2006; Fuglevand 2011). In invertebrates, such as insects, by contrast, the number of motor units is often greatly reduced and all muscle fibers within a single muscle are simultaneously driven by the same or very few motor neurons (Heide 1971; Ikeda 1977; Rheuben and Kammer 1987; Bradacs and Kral 1990; Chakraborty et al. 2015). Besides the number of motor units and noisiness of the neural pathways, precision in motor control also depends on the contractile properties of muscles constituting in the walking, swimming or flying apparatus. Altogether, the signal-to-noise ratio of receptors, their encoding properties, the quality of sensory integration, and the robustness of muscle contraction determine the precision with which animals finally move under both unaffected and externally perturbated locomotor conditions (Faisal et al. 2008; Fuglevand 2011).

**Fig. 1:**
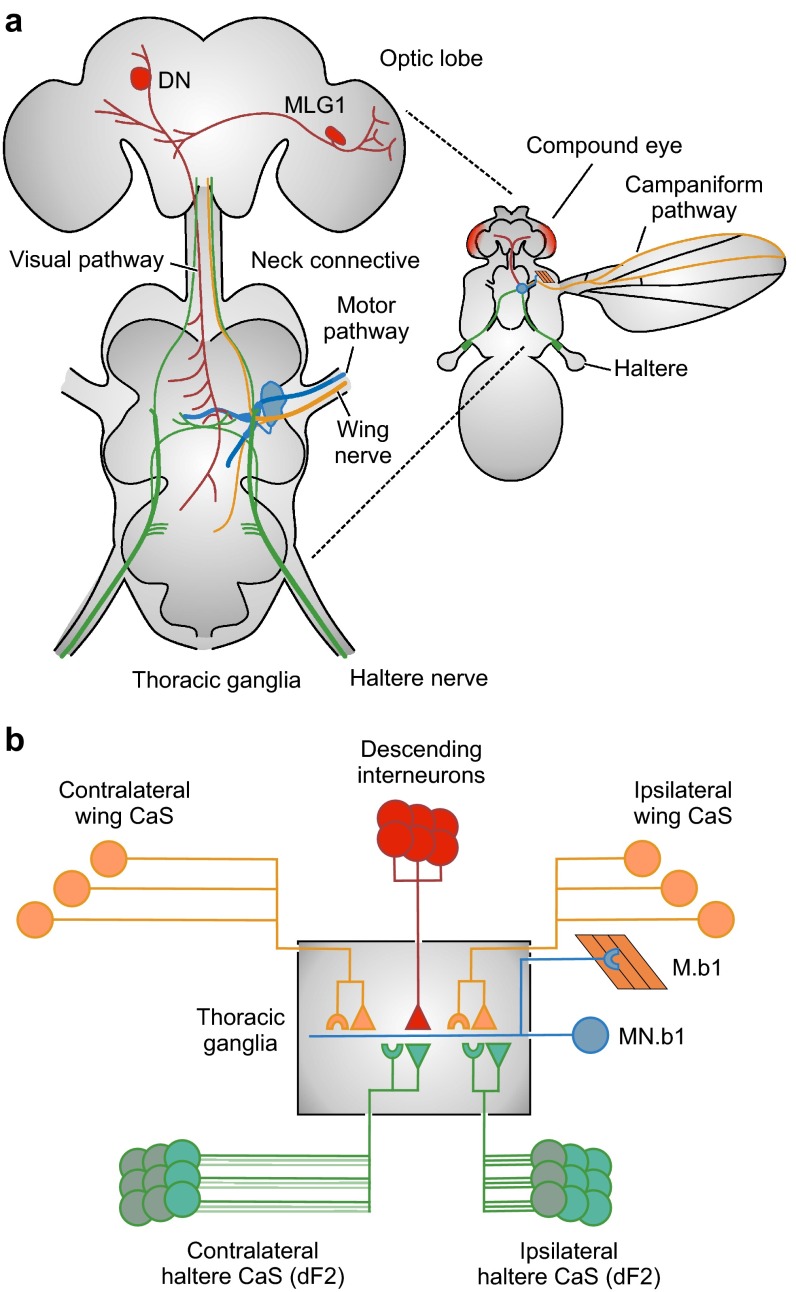
Neural circuitry for flight control in flies, showing input pathways to the ipsilateral basalare 1 flight control muscle (M.b1, Fig. 3). **a** Anatomical and **b** conceptual drawings of neurons that innervate the M.b1 motoneuron (MN.b1, *blue*). Results were taken from various authors. MN.b1 receives ipsi- and contralateral input from descending interneurons (DN, ~50 neurons, *red*) and afferences of mechanoreceptive campaniform sensilla (CaS) located on halteres (*green*) and wings (*orange*). In *Drosophila*, MN.b1 has a disk-shaped soma (15–20 µm diameter), a large axon (7–10 µm diameter) and two stubbly dendritic branches (6–10 µm diameter) with short, thick, unbranched secondary neurites (Trimarchi and Schneiderman 1994). Reciprocal, neurobiotin coupling (*Drosophila*, Trimarchi and Murphey 1997; *Calliphora*, Fayyazuddin and Dickinson 1996) and cobalt-coupling (*Calliphora*, Hengstenberg et al. 1988) reveal monosynaptic electrical synapses of MN.b1 with haltere afferents that co-exist with cholinergic chemical synapses (Trimarchi and Murphey 1997, Fayyazuddin and Dickinson 1996). Each haltere features ~335 CaS in distinct fields. In *Calliphora*, ~110 axons from the haltere’s sensory field *dF2* make synapses on other neurons. Ipsilateral afferents form a calyx-like terminal with thick axonal neurites of MN.b1, bypassing most of the dendritic tree. A subset of *dF2* sensilla projects onto the contralateral MN.b1 (Chan and Dickinson 1996). The wing nerve contains ~900 sensory axons (Heide 1983), constituting electrical and chemical synapses onto MN.b1 close to the synapses of the haltere nerve (Fayyazuddin and Dickinson 1999). MLG1, type 1 male lobula giant neuron coupled to descending neuron (DN, Gronenberg and Strausfeld 1991). *Filled circle* soma of neuron; *filled triangle* electrical synapse; *semicircle* chemical synapse

In particular in flight, the precision of locomotor behavior is highly relevant for heading and body postural control using equilibrium reflexes. Posture stability in walking animals benefits from high friction between body limbs and the ground via ground reaction forces (Dickinson et al. 2000) and swimming animals benefit from hydrodynamic forces owing to the elevated viscosity of water. By contrast, motor precision in flying animals, such as large insects, birds and bats is challenged by comparatively little aerodynamic friction between air and both body and wings (Ellington 1984). The importance of friction in flight, however, depends on body size because flight in small insects relatively suffers more viscous friction than the inertia-dominated flight of birds and bats. Although reduced friction between the animal body and the environment reinforces flight maneuverability and aerial agility, small friction (aerodynamic damping) faces the neuromuscular apparatus of an insect with elevated demands on motor precision for body posture stability and steering (Ristroph et al. 2012). Low frictional damping in flight is thus the key to the extraordinary aerial performance of birds, bats and insects, but at the cost of requiring fast and precise visual and proprioceptive feedback-loop systems (Fig. 1; Hedrick et al. 2007, 2009; Hesselberg and Lehmann 2007; Ramamurti and Sandberg 2007; Cheng et al. 2010).

## Muscle precision in insects

**Fig. 2:**
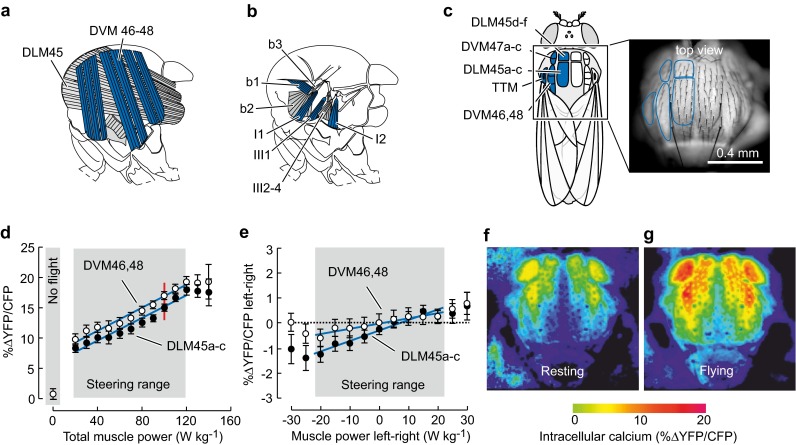
Calcium signaling in the asynchronous indirect flight muscle (A-IFM) of tethered flying fruit flies. **a** Morphology of dorsolongitudinal muscle (DLM) and dorsoventral muscle (DVM) inside the fly thorax (side view). **b** Flight control muscles at the fly’s wing hinge (b1-3, basalare muscles; I1-2 and III2–4, axillary muscles; side view). **c** Top view on thorax and muscle insertion sites. Image shows fluorescence of electrically activated A-IFM in a fruit fly expressing the calcium probe *Cameleon*. **d** Increase in A-IFM intracellular calcium concentration with increasing total A-IFM power output during flight and **e** calcium signaling and muscle power output (left-minus-right body side) during yaw steering (mean ± SD). Calcium levels are calculated from the ratio between the fluorescence of yellow fluorescent protein (YFP) and cyan fluorescent protein (CFP) expressed in *Cameleon*. *Red line* indicates power needed to balance body weight. Data show calcium levels of DVM46, 48 muscles fibers (*open circles*) and DLM45a–c fibers (*closed circles*, Demerec 1965). **f**, **g** Pseudo-color coded image of calcium levels of muscles inside the thorax (*top view*) in resting and flying animals. Figures are adopted from a previous study (Lehmann et al. 2013)

In insects, flight muscle precision and efficacy are crucial for flight control because of high wing stroke frequencies ranging from 5 Hz in some butterflies to 1000 Hz in certain midges (Sotavalta 1947) and the need for relatively small modifications in wing kinematics during maneuvering flight. The fruit fly *Drosophila*, for example, alters wing stroke amplitude by only few angular degrees during maneuvering flight (Götz et al. 1979; Lehmann 1997; Shishkin et al. 2012; Chakraborty et al. 2015; Berthé and Lehmann 2015; Bartussek and Lehmann 2016) and up to a maximum of ~5° during fast saccadic turning about the vertical body axis (Fry et al. 2003). *Drosophila,* moreover, modifies the onset of wing rotation about the longitudinal wing axis at the ventral stroke reversal by less than 70 μs during optomotor yaw stimulation (Dickinson et al. 1993) and also subtly alters wing excursion angle, wing elevation angle and the wing's angle of attack during escape saccades (Muijres et al. 2014, 2015). To support theses tiny modifications in kinematics, dipterans such as *Drosophila* have evolved important strategies to improve the precision of muscle control during wing flapping on a stroke-to-stroke basis. The main strategy is the separation of muscle power for wing flapping from a control system that modifies power transmission to the wings (Fig. 2a, b; Pringle 1968, 1978). From an evolutionary perspective this strategy helps to control wing motion in all insect flight systems based on high-frequency mechanical thoracic oscillators because the number of actions potentials that can modulate muscle force in a 5–10 ms wing stroke cycle is clearly limited.

In dipterans, mechanical power for wing flapping is delivered by the asynchronous, indirect flight muscle (A-IFM) that generates wing motion by an indirect, mechanical linkage between muscle and wings. Wing flapping benefits from the elastic and resonance properties of the thorax shell (Fig. 2a; Pringle 1978; Götz 1983; Heide 1983; Heide et al. 1985; Tu and Dickinson 1996; Dickinson and Tu 1997; Lehmann et al. 2013). Flight control muscles, by contrast, reconfigure the wing hinge of the thoracic oscillator, controlling mechanical properties of the thorax, and thus power transmission from the A-IFM to the flapping wings via the wing hinge (Fig. 2b; Egelhaaf 1989; Lehmann 1997; Balint and Dickinson 2001, 2004; Flick et al. 2001; Wang et al. 2008; Deora et al. 2015). Time-resolved three-dimensional tomographic X-ray microscopy on musculoskeletal mechanisms has recently shown in unmatched detail how flight control muscles in flies are oscillatory stretched and relaxed within each stroke cycle owing to the mechanical movements of wing sclerites (Walker et al. 2014; Mokso et al. 2015). Muscle force generation depends on the exact timing with which the neuronal spikes activate the tissue, whereby the impact of muscle force on wing kinematics continuously changes with changing activation phase. Shifting the timing of the electrical activation of muscles within the stroke cycle is thus a convenient way for the neural system to gradually alter muscle force, which in turn enhances muscle precision control during steering.

A widely neglected parameter that gradually alters muscle force independent of neural activation is heat resulting from the low efficiency between ~5 and ~20%, with which insect flight muscles convert chemical energy into mechanical power (Casey 1981; Stevenson and Josephson 1990; Lehmann and Dickinson 1997; Lehmann 2001; Samejima and Tsubaki 2010; Crespo et al. 2012). Heat production in *Drosophila* helps to boost A-IFM power production at ambient temperatures below 15 °C that otherwise hinder active flight (Lehmann 1999). After take-off, thorax surface temperature of flies increases from ambient temperature to more than 40 °C, changing mechanical power output in a muscle contraction work-loop 3.5-fold compared to the power produced at 20 °C ambient temperature (Gilmour and Ellington 1993b). Elevated temperature causes an increase in both the range with which A-IFM can do oscillatory work and the amount of work per oscillatory cycle (Machin et al. 1962). Thus, in large insects, the precision with which flight muscles deliver power is affected by dorsoventral temperature gradients inside the thorax. For example, the temperature difference in flight muscle fibers of the hawk moth *Manduca* between outer and inner fibers amounts to ~5.6 °C (George and Daniel 2011; George et al. 2012). This temperature gradient produces a mechanical energy gradient in the dorsolongitudinal flight muscles from dorsal to medial, impeding the precision of power control of the entire flight muscle. The animal compensates for this unequal power output by changes in activity of the underlying neural system. Alterations in heat producing grades in muscle power have even been applied to cyborg-like, hybrid insects that use implanted neuromuscular prosthetic devices to decrease the preflight warm-up duration (Bozkurt et al. 2008).

## Motor precision depends on learning and previous flight experience

In most vertebrates, the precision of locomotor control is largely tuned by motor learning and previous experience. In humans it typically takes several years of practice to become skilled in demanding motor tasks and sports disciplines (Hamer et al. 2002), while flight of birds mainly depends on the development of muscles and neurons during maturation, and is thus primarily independent of learning (e.g., Yoda et al. 2004). Nevertheless, birds also learn to fly more efficiently within days or weeks after leaving the nest, increasing flight performance with the number of days since fledging (Yoda et al. 2004). Motor skills in insects, by contrast, are widely recognized as being predominately innate, genetically programmed, fixed-action motor patterns that follow stereotyped rules. An increasing number of studies, however, suggest that experience fine-tunes locomotion to a higher precision. This was shown in walking stick insects (Blaesing and Cruse 2004) and fruit flies (Pick and Strauss 2005) when they encounter and cross gaps, for flight initiation of locusts (Wilson 1961) and the vision-induced landing response in flies (Borst 1990).

**Fig. 3:**
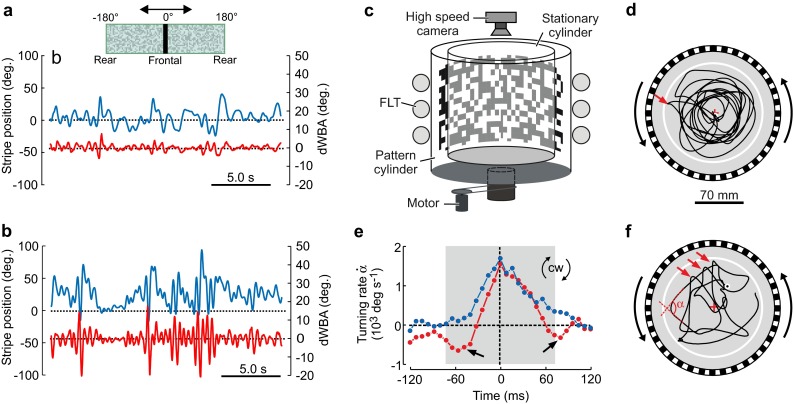
Flight dynamics in tethered and freely flying non-flight-experienced (naïve) and flight-experienced fruit flies (control). **a** Figure shows relative changes in the difference between left and right wing stroke amplitude (*red*, *right scale*) of a flight-experienced fruit fly (control) and the azimuth position (*blue*, *left scale*) of a 30° wide, black, vertical stripe (pictogram) displayed in front of a random-dot background pattern inside a virtual-reality flight simulator (Hesselberg and Lehmann 2007). The animal controls angular velocity and thus position of the stripe by actively changing left and right stroke amplitude. **b** Time traces similar to **a** but derived for a naïve *Drosophila*. Sequences show the flies’ behaviors 100–120 s after flight initiation. **c** Free-flight arena. Single fruit flies emerge in the middle of the arena on an elevated platform and voluntarily initiate flight. A high-speed video camera is triggered when the animal takes off. The motor rotates the random-dot visual environment and three circular fluorescent light tubes (FLT) illuminate the visual pattern from behind. **d**, **f** Typical flight paths (7.9 s flight time) of a control in **d** and a naïve fly in **f**, during clockwise rotation of the visual panorama (angular velocity = 500° s^−1^). **e** Yaw turning rate during clockwise free flight saccades (*red arrows* in **d**, **f**) in naïve (*blue*, *N* = 13 flies) and control (*red*, *N* = 16 flies) fruit flies. Figures are adopted from a previous study (Hesselberg and Lehmann 2009)

Studies on visual and olfactory learning and plasticity of adult *Drosophila* and their underlying brain structures have demonstrated the enormous capacity of this animal to adapt to environmental stimuli (e.g., De Belle and Heisenberg 1994; Dill et al. 1995; Liu et al. 1999; Heisenberg 2015; Giurfa 2015). The flying fruit fly also learns modulations of moments for yaw turning in response to visual motion when trained with heat punishment (Wang et al. 2003). A previous study has shown that self-learning (operant conditioning), a form of motor learning, depends on the activity of protein kinase C (PKC) in many animals and behaviors, including biting in *Aplysia*, song-learning in birds, procedural learning in mice and avoiding behaviors in flying fruit flies (Colomb and Brembs 2016). If flight is deprived within the first 3 days after hatching by raising *Drosophila* in a flat chamber, tethered flying fruit flies employ more corrective steering while heading towards visual objects (Fig. 3a). This behavior results in larger peak-to-peak yaw turning moments than in controls (Fig. 3b; Hesselberg and Lehmann 2009). Untrained, naïve fruit flies reduce their maximum forward speed by ~23% compared to controls and also loose their ability to precisely compensate their flight course for visual perturbations in the environment when flying freely under optomotor conditions (Fig. 3c–f; Hesselberg and Lehmann 2009). The loss in precision in controlling wing motion is also evident in saccadic yaw turning angles, because naïve flies exhibit ~31% larger turning angles (~157°) than adults with previous free flight experience (~108°). Other authors found similar saccadic turning angles for *Drosophila* raised in conventional breeding vials but slightly smaller angles in flies tested in a large free-flight arena (Mronz and Lehmann 2008; Bender and Dickinson 2006b). The fine structure of saccadic turns shows that experienced flies temporarily counter turn prior and after saccades while naïve flies do not, suggesting a subtle impairment in flight control (Fig. 3e). The loss in turning precision in naïve *Drosophila*, however, is not due to an impairment in power generation of the asynchronous, indirect flight muscle because maximum flight muscle force seems to be widely unchanged compared to control flies. This finding runs counter to the idea that a loss in control precision is due to a loss in A-IFM exercise. Muscle exercise may even be unforable in flies because it causes an increase in mortality rate as a result of increased oxidative damage to flight muscles (Yan and Sohal 2000; Magwere et al. 2006).

The reported modifications of wing control in flight-deprived *Drosophila* shift the frequency spectrum of moment-underlying wing stroke amplitudes towards higher values. This indicates an increase in dynamic range within the feedback-loop for flight control. It has thus been suggested that the loss of fine-tuning and locomotor precision in flight of naive *Drosophila* likely reflects a loss of synaptic adaptation in the motor pathway (Hesselberg and Lehmann 2009). Nevertheless, the finding is also consistent with the idea of a loss in control precision with which synchronous, direct flight control muscles fine tune stroke amplitudes during wing flapping (Tu and Dickinson 1996). Similar to other insects, improving the precision of locomotor control in *Drosophila* is thought to be beneficial during aerial escape, for example, to avoid hunting dragonflies that capture freely flying fruit flies by prediction of their flight course (Combes et al. 2012; Mischiati et al. 2014).

## Precision of neural control of the asynchronous indirect flight muscle

Energy acts as a selective pressure on the evolution of locomotor systems. Energetic costs incur by the sensory system owing to the transduction of environmental stimuli into neural activity, information processing of the nervous system, the transport of respiratory gases and fluids, and during muscle contraction (Niven and Laughlin 2008). To determine the impact of energy as a selective pressure, it is significant to know how an organism expends its energy among the various tissues. Studies suggest, for example, that the human brain consumes ~20% and the retina of blowflies (*Calliphora vicina*) ~8% of the animal’s resting metabolism (Clarke and Sokoloff 1999; Howard et al. 1987). The metabolic most active tissue in the animal kingdom is the A-IFM of insects, requiring metabolic power of up to ~2500 W kg^−1^ flight muscle mass in the honey bee (Feuerbacher et al. 2003). Power production by flight muscles is thus a primary factor that limits flapping flight performance in insects (Ellington 1999).

Precise matching of power output produced by A-IFM to the aerodynamic demands during wing flapping is crucial in flying *Drosophila*. Since wing flapping results from vibrations of the thoracic mechanical resonator, mechanical power is a prerequisite of the animal’s ability to alter kinematic parameters, such as stroke frequency and stroke amplitude (Muijres et al. 2014, 2015). This is due to the expected low power output of flight control muscles that may not accommodate the changing power requirements during lift modulation between ~31.8 and ~77.0 W kg^−1^ flight muscle mass or the asymmetrical power requirements between both wings during turning behavior (Lehmann and Dickinson 1997). If the indirect muscles of the thorax provide muscle power in excess to what is actually needed for wing flapping, the wing hinge must destroy waste power to avoid changes in wing kinematics. Otherwise the power leads to unwanted power-driven changes in wing kinematics at the potential cost of changes in heading direction and a decrease in body stability. However, a waste of power leads to a significant degradation of muscle and flight efficiency, harming the biological fitness of *Drosophila* (Casey 1981, 1989; Ellington 1985; Dickinson and Lighton 1995; Lehmann 2001; Lehmann and Pick 2007). Alternatively, if *Drosophila* may not sufficiently provide instantaneous mechanical power, power transmission to the wings may fail when steering muscles reconfigure wing hinge mechanics during flight maneuvers. Precision of A-IFM activation is thus crucial for energetic efficiency, the stability of body posture, and steering performance.

The need for precise control of muscle power in *Drosophila* is evident and faces the animal with a difficult task owing to A-IFM contraction dynamics. Since insect flight requires higher levels of mechanical power than any other form of animal locomotion, the A-IFM of *Drosophila* offers morphological and physiological specializations such as stretch activation and shortening deactivation at an oscillation frequency of more than 200 Hz. Stretch activation describes the transition from a non-force-producing (weekly bound state) cross-bridge-state to a force-producing (strongly bound state) state, termed ‘three-state cross-bridge model’ (Tawada and Kawai 1990; Zhao and Kawai 1993). The transmission of strain to the muscle’s myosin filament (thick filament) likely affects strain-sensitive rate constants of the cross-bridge-cycle, and thus the distribution of cross-bridge-states in A-IFM myofibrils (Granzier and Wang 1993a, b). Previous studies on A-IFM function suggest that the A-IFM’s low spike frequency between 5 and 20 Hz maintains rather constant intramuscular calcium levels during flight (Gilmour and Ellington 1993a; Gordon and Dickinson 2006). Calcium-activated muscle tension, however, is minor in *Drosophila* A-IFM, producing only ~30% of total tetanic contraction by actin-myosin cross-bridge-cycling (Tohtong et al. 1995; Wang et al. 2011). Since the calcium-activated isometric force component accounts for only fractions of the power required to sustain active flight, stretch activation due to ~1.0–2.5% alterations in fiber length increases the number of cross-bridge cycles of the calcium-activated muscle (Tohtong et al. 1995). This cross-bridge recruitment leads to a delayed threefold to fourfold increase in force during muscle shortening, and hence increases muscle work and power generation within each stroke cycle (Josephson et al. 2000; Swank 2012). Shortening deactivation decreases force levels during lengthening by decreasing the number of cross-bridges, which attenuates muscle stiffness when the fibers undergo their cyclic shortening–lengthening cycle at a relatively constant calcium levels (Pringle 1978; Peckham et al. 1992; Thomas and Thornhill 1995; Josephson and Syme 2001; Moore 2006).

Experimental studies recently questioned the simple picture on power control by A-IFM stretch-activation, focusing on the calcium-induced power production mediated by the troponin-tropomyosin complex as part of the thin filament components of A-IFM (Gordon and Dickinson 2006; Lehmann et al. 2013). This complex typically regulates cross-bridge binding and contraction in striated muscles such as flight control muscles, in which Troponin C (TnC) acts as the calcium-sensor, triggering contraction. By contrast, genes controlling *Drosophila* A-IFM development express two TnC isoforms: the isoform TnC4 (DmTnC4) is sensitive to stretch-activation owing to the mechanic resonance of the thoracic box, while the second isoform TnC41C (DmTnC1) is sensitive to calcium, producing muscle power output as a function of intramuscular calcium levels, similar to striated muscles (Wang et al. 2011). The isoforms yield a molar DmTnC4:DmTnC1 ratio of approximately 10:1 (Qiu et al. 2005; Kržič et al. 2010). The presence of both stretch-sensing and calcium-sensing troponin isoforms in the same muscle might indicate an evolutionary advantage of this hybrid expression pattern for contraction control. In *Drosophila*, the exact function of isoforms TnC4 and TnC41C for muscle power control is not yet understood but offers the option to precisely match power output of the A-IFM by variation in calcium activation during flight. Controlling TnC41C by precise alterations in muscle spike frequency via the neural pathways may compensate for insufficient power control owing to stretch-activation and highlights the significance of spike frequency and temporal precision inside the various A-IFM fibers of *Drosophila* (Gordon and Dickinson 2006; Heide et al. 1985).

The idea of precise power control by variation in calcium levels contrasts the calcium-switching hypothesis and is supported by at least three studies. The first in vitro study demonstrated that increasing calcium inside A-IFM leads to an increasing power output during cyclic stretching in fibers using work-loop technique (Wang et al. 2011). The latter investigation was conducted in isolated, skinned muscle fibers mounted in a force rack for tension measurements and showed a steep increase in positive muscle power with increasing calcium concentration (pCa) within a small range of calcium from pCa = 5.0 to pCa = 5.8. Above and below this threshold, forces were constantly minimum or maximum, respectively. In the second study, Gordon and Dickinson (Gordon and Dickinson 2006) recorded the spike frequency of specific A-IFM fibers during maneuvering flight of *Drosophila*, while the tethered animal changed its instantaneous locomotor capacity in response to moving visual stimuli. The measured spike frequency of up to ~20 Hz was subsequently applied as trains of electrical pulses for stimulation of the muscle tissue in vivo. Expressing the calcium indicator *Cameleon* inside the muscle fibers, the authors were able to show a twofold linear increase of calcium within the working range of the thoracic flight motor. Although both studies highlighted that muscle spike frequency alters calcium activation via the troponin isoform TnC4, they could not demonstrate how calcium signaling is spatially distributed between various fibers of A-IFM. In particular, turning flight requires an asymmetrical distribution of flight muscle power due to asymmetrical aerodynamic drag on the two flapping wings. A flight system that supports these asymmetries on the level of neural control of power muscles is thus beneficial for power balancing. This aspect of flight control in fruit flies was investigated by in vivo measurements, simultaneously scoring calcium-dependent fluorescence by *Cameleon* in several DLM and DVM muscle fibers during vision-induced maneuvering flight (Lehmann et al. 2013). The study demonstrated a highly linear relationship between intramuscular calcium concentration and muscle power production with regression coefficients (*R* ^2^ values) ranging from ~0.95 in DLM to ~0.97 in fibers of the DVM and at muscle power output between ~20 and ~120 W kg^−1^ flight muscle mass. Precise A-IFM power adjustments occur through bilateral control of calcium levels between the two thoracic segments within a comparatively small range of intramuscular calcium levels from ~56 to ~79 nM (Lehmann et al. 2013). The spatial A-IFM activation pattern thereby shows that a unilateral increase in calcium is correlated with an increase in stroke amplitude, and thus an increase in aerodynamic wing drag. This likely means that the neural drive to A-IFM fibers controls power for each body side and half stroke, dependent on flight commands induced by the fly’s compound eyes.

## Timing of flight control muscle activation by proprioceptive feedback

What we experience as flight behavior of *Drosophila* reflects the output of a complex high-speed feedback cascade that turns sensory information into locomotor forces, linking muscle tissue activation to the various neural pathways in the fly (Borst and Haag 2002; Fotowat et al. 2009; Borst et al. 2010). The complexity of this feedback control loop, including all its facets, is still under investigation and understanding the integration process of signals coming from the compound eyes, ocelli, antennae, campaniform sensilla on wings and body, and the gyroscopic halteres remains a challenge. Thus, there is a continuing debate on which sensory feedback is needed for flight and how sensory information, for example, from halteres and eyes are temporally encoded to provide the desired precision for body posture, flight course control, and equilibrium reflexes (Sherman and Dickinson 2003, 2004; Frye and Dickinson Michael 2004; Bender and Dickinson 2006a; Huston and Krapp 2009; Frye 2010; Bartussek et al. 2013; Bartussek and Lehmann 2016). A fairly comprehensive review on sensory control of insect flight was previously published by Taylor and Krapp (2007).

Precise muscle control in flies requires an exact timing of muscle spike initiation within the stroke cycle because the efficacy of flight control muscles on wing kinematics varies with changing activation phase. This was demonstrated by simultaneous electrophysiological recordings of muscle activity and wing kinematics (Lehmann and Götz 1996; Tu and Dickinson 1994, 1996; Heide and Götz 1996). Flies may thus gradually control wing kinematics without alterations in muscle spike frequency. Preferred muscle activation phases in flies have been reported for flight control muscles basalare 1 and 2, axillare I1, axillare III1-4 (Heide 1979; Lehmann and Götz 1996; Balint and Dickinson 2001) and fibers of A-IFM (Spüler and Heide 1978; Heide et al. 1985). Muscle activation phases may widely vary during flight and are often temporally distributed over the entire wing stroke cycle, which is shown for multiple muscles in the blowfly *Calliphora* (Balint and Dickinson 2001). Activation phases, however, consistently shift during yaw maneuvers, as shown for spikes of the basalare 1 muscle during turning flight in *Musca* (~63°; Egelhaaf 1989), *Calliphora* (~26°; Tu and Dickinson 1996; Balint and Dickinson 2001), and *Drosophila* (~39°; Heide and Götz 1996). Phase shifts are also found in other insects such as moth, in which the left–right pairs of dorsolongitudinal and ventral muscles precisely fire within 0.5–0.6 ms of each other. Data show that this timing difference typically increases to ~8 ms during yaw turning of the animal (Sponberg and Daniel 2012; Springthorpe et al. 2012).

Since their first detailed investigation (Pringle 1948; Nalbach 1993, 1994; Nalbach and Hengstenberg 1994), the halteres of flies are known to be significant for muscle activation because of their fast feedback within the 5–10 ms wing stroke cycle and their capability to time motoneuron spike initiation of flight control muscles (Fayyazuddin and Dickinson 1999; Fox et al. 2010; Fox and Daniel 2008). It is the gating-like behavior of motoneurons that enables the flight apparatus to phase-couple muscle spike initiation with the haltere stroke cycle (Huston and Krapp 2009). In blowflies, this neural gating is accomplished by the campaniform field *dF2* at the haltere’s basis that provides strong input via electrical synapses to the motor neuron driving the first basalare control muscle (Fig. 1b; Chan and Dickinson 1996; Fayyazuddin and Dickinson 1996). According to this concept, alterations in visually induced wing motion result from changes in halteres movements, whereby these changes are mediated by activation of haltere control muscles (Chan et al. 1998). The main benefit of this ‘neural rerouting’ would be that haltere-mediated body stability reflexes and vision-controlled flight reside in the same neuromuscular network, without facing the problem of a functional interference between equilibrium reflex control by haltere and flight heading control owing to vision and olfactory system (Dickinson et al. 2000; Sherman and Dickinson 2004; Bender and Dickinson 2006a; Frye 2007).

**Fig. 4:**
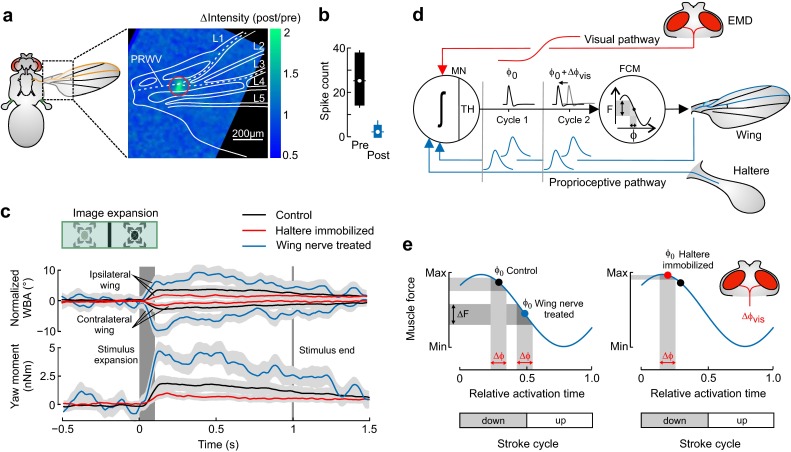
Envelope of wing kinematics and steering precision depends on proprioceptive feedback in tethered flying fruit flies. **a** Relative change in fluorescence intensity (post–pre-application stimulus ratio) plotted in pseudo-color after local (*red ring*) application of a laser pulse, attenuating wing nerve signaling. The wing structures are retraced from bright-field microscopy and the wing nerve is shown according to anatomical studies (*white*, *dashed line*). PRWV, proximal radial wing vein. **b** Wing nerve spike counts owing to repetitive mechanical stimulation of the wing’s campaniform sensilla, prior (pre) and posterior (post) laser treatment of the wing nerve (*N* = 6). **c** Wing kinematics and instantaneous yaw moment in tethered flies, flying in a flight simulator and during vision-triggered escape saccades (radial expansion pattern, pictogram). The pattern vanishes ~1 s after stimulus onset from the panorama (*grey line*). Traces show mean responses of ipsi- (stimulus side) and contralateral wing that were offset-normalized by subtraction of mean pre-saccadic kinematics (*top*) and yaw moment (*bottom*). *Light grey* area around each data trace indicates one fifth of the standard deviation. *Black*, intact controls (*N* = 24 flies); *red* flies with bilaterally immobilized halteres (*N* = 17); and *blue* animals with laser-ablated wing nerves (*N* = 5 flies). **d** Hypothetical feedback loop for activation timing of a flight control muscle (steering muscle). Strain-sensitive campaniform sensilla on wings and halteres produce neural spikes (*blue*) or volleys of spikes at specific times of the wing stroke cycle (phase-coupled activation). The elementary motion detector (EMD) of the fly’s compound eye converts visual motion into graded potentials (*red*) that are transmitted via descending visual interneurons to the thoracic ganglion (visual pathway). The three inputs are integrated by a control muscle motoneuron (MN; ʃ, integration process), generating a single muscle action potential at the neuron’s threshold (TH) and time ϕ_0_ (activation phase) in each stroke cycle (cycle 1). A change in visual signaling alters the motoneuron’s membrane potential, and thus delays or advances spike timing (Δϕ_0_) by alterations of depolarization time (cycle 2). Force (F) of the flight control muscle (FCM) changes depending on spike timing because of the muscle’s nonlinear ‘force-phase’ curve (Lehmann and Götz 1996; Tu and Dickinson 1996, 1994; Heide and Götz 1996). The changing work finally leads to changes in transmission efficacy, and thus wing motion. **e** Hypothetical schematics of muscle force control by temporal changes in muscle spike generation. Mean activation phase (ϕ_0_) in control (*black dot*), wing nerve ablated (*blue dot*) and haltere treated flies (*red dot*) determines the range of muscle force generation during vision-induced phase shifts (Δϕ_vis_, *red*). Force envelope is smaller in halteres treated and larger in wing nerve treated flies, compared to controls. Figures are partly adopted from a previous study (Bartussek and Lehmann 2016)

More recent behavioral studies in *Drosophila*, however, questioned this conventional rerouting pathway of visual information for wing control, suggesting a direct neural pathway between the visual system and motoneurons of flight control muscles (Mureli and Fox 2015; Bartussek and Lehmann 2016). The studies also suggest a sensory integration mechanism that conceptually represents a neural local sensory feedback circuitry for motor control similar to what has been found in stick insects (Büschges and Gruhn 2008), cats (Ekeberg and Pearson 2005), and humans (Yang and Gorassini 2006). This local circuitry provides feedback with little delay within a single stroke cycle. Nevertheless, the finding of a direct, functional connection between visual system and motoneurons is less surprising because intracellular recordings combined with dye filling showed that in Diptera more than 50 pairs of visual motion-sensitive descending neurons from the brain terminate bilaterally in superficial pterothoracic neuropils at the level of motoneurons (Fig. 1a; Gronenberg and Strausfeld 1991). Motion-sensitive descending neurons that respond to yaw, pitch and roll movements of the fly, for example, provide segmental collaterals to neuropils containing motoneurons of flight control and neck muscles (Strausfeld and Gronenberg 1990). A study on male flesh flies *Sarcophaga* showed that the descending visual interneurons DNDC3-6a are dye-coupled to motoneurons of the two most prominent steering muscles b1 and b2 (Gronenberg and Strausfeld 1991). The apparent absence of vision-evoked electrical responses in flight control muscles that has been reported by Chan and colleagues (Chan et al. 1998) in *Calliphora* may thus be explained by the motoneuron’s gating process in the in vitro preparation. The latter view is also supported by electrophysiological studies on visual interneurons and neck muscles of flies. These data show that visual stimulation induces spiking of neck muscle motoneurons only during locomotor activity (Haag et al. 2010) owing to an increase in gain of visual interneurons (Maimon et al. 2010; Rosner et al. 2010). A potential physiological mechanism for integration of non-phasic visual information and phasic proprioceptive feedback from halteres and wings is currently under debate (Fig. 4; Bartussek and Lehmann 2016) and based on observations on graded, non-spiking responses of visual interneurons (Haag et al. 2007) and sustained, subthreshold depolarization of neck motoneurons following visual stimulation in flies (Huston and Krapp 2009).

Besides visual pathways and feedback from the gyroscopic halteres, spike timing of flight control muscles also depends on neural projections from mechanoreceptors located on both wings (Cole and Palka 1982; Gnatzy et al. 1987; Fayyazuddin and Dickinson 1999). Wing mechanoreceptors control body posture and spike timing in moth (Dickerson et al. 2014) and flies (Heide 1979, 1983; Balint and Dickinson 2001), providing feedback on wing loading (Hengstenberg 1991) and wing deformation caused by the travel of torsional waves over the wing surface (Dickinson 1990). The functional role of wing mechanoreceptors for flight control, however, is not well understood (Taylor and Krapp 2007). A recent study suggests that proprioceptive feedback from wing mechanoreceptors acts antagonistically to the feedback provided by the halteres (Fig. 4; Bartussek and Lehmann 2016). This was shown in tethered flying fruit flies, in which either the wing nerve or haltere feedback signaling was abolished. In response to moving (fixation response) or expanding visual stimuli (escape response) displayed in a flight simulator, fruit flies bilaterally vary their stroke amplitudes dependent on proprioceptive feedback. At flight conditions (aerodynamic damping) similar to those expected for free flight, a reduction in feedback from the wings’ mechanoreceptors leads to an increase in kinematic envelope, i.e., the difference between minimum and maximum wing flapping amplitude, from ~19° in controls to ~36° (Fig. 4c; Hesselberg and Lehmann 2007). By contrast, an attenuation of feedback signaling from the halteres leads to a decrease in kinematic envelope from ~19° to ~10° although both pathways provide excitatory synaptic input to the flight muscle motoneuron (Fayyazuddin and Dickinson 1999). This puzzling result may not easily be explained.

A possible explanation for the above finding is that compression and expansion of kinematic envelope result from a change in the efficacy with which flight control muscles alter wing stroke amplitude (Bartussek and Lehmann 2016; Tu and Dickinson 1994). It has previously been shown that transmission efficacy of flight control muscles sinusoidally depends on spike activation phase (‘force-phase’ curve). Thus, different mean spike activation phases of wing and haltere signaling within the stroke cycle may produce different setpoints on the 'force-phase' curve and, eventually, also different dependencies between muscle force and visual signaling (Fig. 4d, e). The direct link between spike timing, muscle force and wing kinematics has previously been demonstrated in M.b1 by scoring spike timing and in M.b2 by electrical stimulation at various times of the stroke cycle (Tu and Dickinson 1994; Lehmann and Götz 1996). In this light, the cyclic stretching and the corresponding cyclic changes in muscle tension during wing flapping is a prerequisite for the flight system to convert changes in spike timing into biomechanical alterations of wing motion. A preliminary, numerical model on thoracic spike initiation in *Drosophila* also validates the assumption that graded visual signals are able to shift the timing of spike generation at the level of motoneurons, in turn changing kinematic efficacy during vision-controlled flight. The modeling also suggests that the two proprioceptive modalities are finely balanced with the biomechanical properties of flight control muscles, stabilizing spike activation in a narrow temporal phase band of the stroke cycle and depending on the animal’s current locomotor state.

A remarkable feature of the sensorimotor circuitry for flight is the elevated number of electrical synapses. Wing steering muscles and neck muscles are typically supplied by numerous motion-sensitive, descending interneuron via electrical synapses (Strausfeld and Gronenberg 1990; Gronenberg and Strausfeld 1990). The same holds for the haltere and wing nerves, both constituting electrical and chemical synapses on flight muscle motoneurons (Fig. 1; Trimarchi and Murphey 1997; Fayyazuddin and Dickinson 1996, 1999). There is an ongoing controversy on the benefits of this unusual neural design. Chemical synapses often show fatigue at high-frequency stimulation owing to a depletion of presynaptic vesicles (Zucker 1989). Studies on synaptic transmission of wing and haltere nerves in the blowfly *Calliphora* widely confirmed this assumption for frequencies that are close to normal wingbeat frequency of ~130 Hz (Fayyazuddin and Dickinson 1996, 1999). By contrast, electrical synapses are typically less sensitive to repeated stimulation (Jaslove and Brink 1987) and the use of electrical synapses in a high-frequency motor circuitry appears to be obvious. It is thus surprising that in blowflies chemical and electrical components of the MN.b1′s excitatory postsynaptic potential (EPSP) show a frequency-dependent decrement in size, although the electrical component of the haltere nerve is larger and apparently more robust (Fayyazuddin and Dickinson 1999).

An alternative explanation for the dominance of electrical synapses is that timing precision of MN.b1 spikes likely increases with decreasing EPSP length. For example, the rise time of the electrical component of MN.b1-EPSP in blowflies is ~0.6 ms, compared to ~1.6 ms of the chemical component. This 1 ms difference is significant because it represents a relatively large fraction of the ~5 and ~6.7 ms wing stroke period in *Drosophila* and *Calliphora*, respectively. Since the combined EPSP is significantly shorter following haltere nerve stimulation (~1.1 ms, time at half width response) compared to wing nerve stimulation (~3.8 ms), halteres likely deliver more accurate timing cues for spike initiation in the motoneuron than wing mechanoreceptors. This conclusion is also consistent with the finding that the temporal jitter in MN.b1-spiking during repeated haltere stimulation is less than during wing nerve stimulation (Fayyazuddin and Dickinson 1999).

## Conclusions: precision of muscle activation

Exposed to aerial predation and little aerodynamic friction, flying insects such as *Drosophila* have evolved unique adaptations of their thoracic flight apparatus. The precise control of muscle mechanical forces is key for the extraordinary flight performance in these animals and evolution has selected several unusual mechanisms for flight force control. The expression of stretch- and calcium sensitive muscle troponins, the development of neural circuitries that stabilize muscle activation patterns in narrow temporal phase bands and the fusion of multimodal information from the fly’s ocelli and compound eyes at the level of descending neurons are prominent achievements (Parsons et al. 2010). Recent work also affirms the principle of reafference for flight control on a cellular level, showing that lobula plate tangential neurons in *Drosophila* suppress the perception of self-generated visual motion during body turns (Kim et al. 2015). This suppression potentially allows spontaneous body saccades in flight by attenuation of optomotor reflexes. The required efference copies are thought to be either internally generated signals or proprioceptive sensory feedback from the turn mediated by the halteres.

In general, studies on how graded visual signaling from the compound eyes is fused with spiking proprioceptive feedback from halteres and wings tackle not only principles of neural coding and timing but address fundamental problems of sensory integration processes. It appears that fast and precise timing of flight muscle activation is achieved by sensory integration at the level of single motoneurons that circumvents unwanted temporal delays in spike transmission, unavoidably occurring by the employment of more complex neural circuitries residing in the thoracic ganglia and the central brain. While haltere feedback varies depending on Coriolis forces caused by body movements, wing mechanoreceptors encode changes in aerodynamic and inertial forces acting locally on the wing during flapping motion. According to the feedback of these sensory systems, the proprioceptive circuitry in *Drosophila* dynamically changes the preferred phase value from stroke-to-stroke, and consequently, the instantaneous force-phase gain of flight control muscles. Despite solely providing excitatory synaptic input to flight muscle motoneurons, sensory signals from wings and halteres cause opposing effects on visuomotor gain and flight control precision, implying that effective flight control is due to a complex interplay between muscle-specific, nonlinear power generation and precise timing cues provided by mechanoreceptors. In conclusion, the neural control processes for flight muscle activation during flight of the fruit fly require temporal precision within fractions of a millisecond, which makes *Drosophila* an ideal research model for high-performance locomotor systems (Frye 2010) and also a paradigm for man-made, biomimetic aerial robotic systems (Stafford 2007).

## References

[CR1] Balint CN, Dickinson MH (2001). The correlation between wing kinematics and steering muscle activity in the blowfly *Calliphora vicina*. J Exp Biol.

[CR2] Balint CN, Dickinson MH (2004). Neuromuscular control of aerodynamic forces and moments in the blowfly, *Calliphora vivina*. J Exp Biol.

[CR3] Bartussek J, Lehmann F-O (2016). Proprioceptive feedback determines visuomotor gain in *Drosophila*. R Soc Open Sci.

[CR4] Bartussek J, Mutlu AK, Zapotocky M, Fry SN (2013). Limit-cycle-based control of the myogenic wingbeat rhythm in the fruit fly *Drosophila*. J R Soc Interface.

[CR5] Bender JA, Dickinson MH (2006). A comparison of visual and haltere-mediated feedback in the control of body saccades in *Drosophila melanogaster*. J Exp Biol.

[CR6] Bender JA, Dickinson MH (2006). Visual stimulation of saccades in magnetically tethered *Drosophila*. J Exp Biol.

[CR7] Berthé R, Lehmann F-O (2015). Body appendages fine-tune posture and moments in freely manoeuvring fruit flies. J Exp Biol.

[CR8] Blaesing B, Cruse H (2004). Stick insect locomotion in a complex environment: climbing over large gaps. J Exp Biol.

[CR9] Borst A (1990). How do flies land?. Bioscience.

[CR10] Borst A, Haag J (2002). Neural networks in the cockpit of the fly. J Comp Physiol A.

[CR11] Borst A, Haag J, Reiff DF (2010). Fly motion vision. Annu Rev Neurosci.

[CR12] Bozkurt A, Lal A, Gilmour R (2008) Electrical endogenous heating of insect muscles for flight control. In: Engineering in medicine and biology society, 2008. EMBS 2008. 30th Annual international conference of the IEEE, 2008. IEEE, pp 5786–5789 10.1109/IEMBS.2008.4650529 19164032

[CR13] Bradacs H, Kral K (1990). Innervation of an insect asynchronous flight muscle as seen with scanning electron microscopy. Z Mikros Anat Fosch.

[CR14] Büschges A, Gruhn M (2008). Mechanosensory feedback in walking: from joint control to locomotor patterns. Adv Insect Physiol.

[CR15] Casey TM (1981). A comparison of mechanical and energetic estimates of flight cost for hovering sphinx moths. J Exp Biol.

[CR16] Casey TM, Goldsworthy GJ, Wheeler CH (1989). Oxygen consumption during flight. Insect flight.

[CR17] Chakraborty S, Bartussek J, Fry SN, Zapotocky M (2015). Independently controlled wing stroke patterns in the fruit fly *Drosophila melanogaster*. PLoS One.

[CR18] Chan WP, Dickinson MH (1996). Position-specific central projections of mechanosensory neurons on the haltere of the blow fly, Calliphora vicina. J Comp Neurol.

[CR19] Chan WP, Prete F, Dickinson MH (1998). Visual input to the efferent control system of a fly’s “gyroscope”. Science.

[CR20] Cheng B, Fry SN, Huang Q, Deng X (2010). Aerodynamic damping during rapid flight maneuvers in the fruit fly *Drosophila*. J Exp Biol.

[CR21] Clarke JB, Sokoloff L, Siegel GJ, Agranoff BW, Albers RW, Fisher SK, Uhler MD (1999). Circulation and energy metabolism of the brain. Basic neurochemistry.

[CR22] Cole ES, Palka J (1982). The pattern of campaniform sensilla on the wing and haltere of *Drosophila melanogaster* and several of its homeotic mutants. J Embryol Exp Morph.

[CR23] Colomb J, Brembs B (2016). PKC in motorneurons underlies self-learning, a form of motor learning in *Drosophila*. Peer Rev Open Access.

[CR24] Combes SA, Rundle DE, Iwasaki JM, Crall JD (2012). Linking biomechanics and ecology through predator-prey interactions: flight performance of dragonflies and their prey. J Exp Biol.

[CR25] Crespo JG, Goller F, Vickers NJ (2012). Pheromone mediated modulation of pre-flight warm-up behavior in male moths. J Exp Biol.

[CR26] De Belle JS, Heisenberg M (1994). Associative odor learning in *Drosophila* abolished by chemical ablation of mushroom bodies. Science.

[CR27] Demerec M (1965). Biology of *Drosophila*.

[CR28] Deora T, Singh AK, Sane SP (2015). Biomechanical basis of wing and haltere coordination in flies. PNAS.

[CR29] Dickerson BH, Aldworth ZN, Daniel T (2014). Control of moth flight posture is mediated by wing mechanosensory feedback. J Exp Biol.

[CR30] Dickinson MH (1990). Comparison of encoding properties of campaniform sensilla on the fly wing. J Exp Biol.

[CR31] Dickinson MH, Lighton JRB (1995). Muscle efficiency and elastic storage in the flight motor of *Drosophila*. Science.

[CR32] Dickinson MH, Tu MS (1997). The function of dipteran flight muscle. Comp Biochem Physiol A.

[CR33] Dickinson MH, Lehmann F-O, Götz KG (1993). The active control of wing rotation by *Drosophila*. J Exp Biol.

[CR34] Dickinson MH, Farley CT, Full RJ, Koehl MAR, Kram R, Lehman S (2000). How animals move: an integrative view. Science.

[CR35] Dill M, Wolf R, Heisenberg M (1995). Behavioral analysis of *Drosophila* landmark learning in the flight simulator. Learn Mem.

[CR36] Egelhaaf M (1989). Visual afferences to flight steering muscles controlling optomotor responses of the fly. J Comp Physiol A.

[CR37] Ekeberg Ö, Pearson KG (2005). Computer simulation of stepping in the hind legs of the cat: an examination of mechanisms regulating the stance-to-swing transition. J Neurophysiol.

[CR38] Ellington CP (1984). The aerodynamics of hovering insect flight. IV. Aerodynamic mechanisms. Phil Trans R Soc Lond B.

[CR39] Ellington CP (1985). Power and efficiency of insect flight muscle. J Exp Biol.

[CR40] Ellington CP (1999). The novel aerodynamics of insect flight: applications to micro-air vehicles. J Exp Biol.

[CR41] Faisal AA, Selen LPJ, Wolpert DM (2008). Noise in the nervous system. Nature.

[CR42] Fayyazuddin A, Dickinson MH (1996). Haltere afferents provide direct, electrotonic input to a steering motor neuron of the blowfly, *Calliphora*. J Neurosci.

[CR43] Fayyazuddin A, Dickinson MH (1999). Convergent mechanosensory input structures the firing phase of a steering motor neuron in the blowfly, *Calliphora*. J Neurophysiol.

[CR44] Feuerbacher E, Fewell JH, Roberts SP, Smith EF, Harrison JF (2003). Effects of load type (pollen or nectar) and load mass on hovering metabolic rate and mechanical power output in the honey bee *Apis mellifera*. J Exp Biol.

[CR45] Flick KC, Tu MS, Daniel TL (2001). Flight control by steering muscles in *Manduca sexta*. Am Zool.

[CR46] Fotowat H, Fayyazuddin A, Bellen HJ, Gabbiani F (2009). A novel neuronal pathway for visually guided escape in *Drosophila melanogaster*. J Neurosci.

[CR47] Fox JL, Daniel TL (2008). A neural basis for gyroscopic force measurement in the halteres of *Holorusia*. J Comp Physiol A.

[CR48] Fox JL, Fairhall AL, Daniel TL (2010). Encoding properties of haltere neurons enable motion feature detection in a biological gyroscope. PNAS.

[CR49] Fry SN, Sayaman R, Dickinson MH (2003). The aerodynamics of free-flight maneuvers in *Drosophila*. Science.

[CR50] Frye MA (2007). Behavioral neurobiology: a vibrating gyroscope controls fly steering maneuvers. Curr Biol.

[CR51] Frye MA (2010). Multisensory systems integration for high-performance motor control in flies. Curr Opin Neurobiol.

[CR52] Frye MA, Dickinson Michael H (2004). Closing the loop between neurobiology and flight motor. Curr Opin Neurobiol.

[CR53] Fuglevand AJ (2011). Mechanical properties and neural control of human hand motor units. J Physiol.

[CR54] George NT, Daniel TL (2011). Temperature gradients in the flight muscles of *Manduca sexta* imply a spatial gradient in muscle force and energy output. J Exp Biol.

[CR55] George N, Sponberg S, Daniel T (2012). Temperature gradients drive mechanical energy gradients in the flight muscle of *Manduca sexta*. J Exp Biol.

[CR56] Gilmour KM, Ellington CP (1993). In vivo muscle length changes in bumblebees and the in vitro effects on work and power. J Exp Biol.

[CR57] Gilmour KM, Ellington CP (1993). Power output of glycerinated bumblebee flight muscle. J Exp Biol.

[CR58] Giurfa M (2015). Learning and memory in insects. Wiley Interdiscip Rev Cogniti Sci.

[CR59] Gnatzy W, Grünert U, Bender M (1987). Campaniform sensilla of *Calliphora vicina* (Insecta, *Diptera*): I. topography. Zoomorphology.

[CR60] Gordon S, Dickinson MH (2006). Role of calcium in the regulation of mechanical power in insect flight. PNAS.

[CR61] Götz KG, Nachtigall W (1983). Bewegungssehen und Flugsteuerung bei der Fliege *Drosophila*. BIONA-report 2.

[CR62] Götz KG, Hengstenberg B, Biesinger R (1979). Optomotor control of wing beat and body posture in *Drosophila*. Biol Cybern.

[CR63] Granzier HL, Wang K (1993). Interplay between passive tension and strong and weak binding cross-bridges in insect indirect flight muscle: a functional dissection by gelsolin-mediated thin filament removal. J Gen Physiol.

[CR64] Granzier HL, Wang K (1993). Passive tension and stiffness of vertebrate skeletal and insect flight muscles: the contribution of weak cross-bridges and elastic filaments. Biophys J.

[CR65] Gronenberg W, Strausfeld NJ (1990). Descending neurons supplying the neck and flight motor of diptera: physiological and anatomical characteristics. J Comp Neurol.

[CR66] Gronenberg W, Strausfeld NJ (1991). Descending pathways connecting the male-specific visual system of flies to the neck and flight motor. J Comp Physiol A.

[CR67] Haag J, Wertz A, Borst A (2007). Integration of lobula plate output signals by DNOVS1, an identified premotor descending neuron. J Neurosci.

[CR68] Haag J, Wertz A, Borst A (2010). Central gating of fly optomotor response. PNAS.

[CR69] Hamer KC, Schreiber E, Burger J, Schreiber EA, Burger J (2001). Breeding biology, life histories, and life history-environment interactions in seabirds. Biology of marine birds.

[CR70] Hedrick TL, Usherwood JR, Biewener AA (2007). Low speed maneuvering flight of the rose-breasted cockatoo (*Eolophus roseicapillus*). II. Inertial and aerodynamic reorientation. J Exp Biol.

[CR71] Hedrick TL, Cheng B, Deng X (2009). Wingbeat time and the scaling of passive rotational damping in flapping flight. Science.

[CR72] Heide G (1971). Die Funktion der nicht-fibrillären Flugmuskeln bei der Schmeißfliege *Calliphora*. Teil I: lage, Insertionsstellen und Innervierungsmuster der Muskeln. Zool Jb Abt Allg Zool Physiol.

[CR73] Heide G (1979). Proprioceptive feedback dominates the central oscillator in the patterning of the flight motoneuron output in *Tipula* (*Diptera*). J Comp Physiol A.

[CR74] Heide G, Nachtigall W (1983). Neural mechanisms of flight control in *Diptera*. BIONA-report 2.

[CR75] Heide G, Götz KG (1996). Optomotor control of course and altitude in *Drosophila* is achieved by at least three pairs of flight steering muscles. J Exp Biol.

[CR76] Heide G, Spüler M, Götz KG, Kamper K, Wendler G (1985). Neural control of asynchronous flight muscles in flies during induced flight manoeuvers. Insect Locomotion.

[CR77] Heisenberg M (2015). Outcome learning, outcome expectations, and intentionality in *Drosophila*. Learn Mem.

[CR78] Hengstenberg R (1991). Stabilisierende Kopfbewegungen bei der Schmeißfliege *Calliphora erythrocephala*. Zool Jb Abt Allg Zool Physiol.

[CR79] Hengstenberg R, Hausen K, Hengstenberg B (1988) Cobalt pathways from haltere mechanoreceptors to inter- and motor neurons controlling head posture and flight steering in the blowfly *Calliphora*. In: Elsner N, Barth F (eds) 16th Göttingen neurobiology conference, Göttingen, 1988. Thieme, p 129

[CR80] Hesselberg T, Lehmann F-O (2007). Turning behaviour depends on frictional damping in the fruit fly *Drosophila*. J Exp Biol.

[CR81] Hesselberg T, Lehmann F-O (2009). The role of experience in flight behaviour of *Drosophila*. J Exp Biol.

[CR82] Howard J, Blakeslee B, Laughlin SB (1987). The intracellular pupil mechanism and photoreceptor signal: noise ratios in the fly Lucilia cuprina. Proc R Soc Lond B Biol Sci.

[CR83] Huston SJ, Krapp HG (2009). Non-linear integration of visual and haltere inputs in fly neck motor neurons. J Neurosci.

[CR84] Ikeda K, Hoyle G (1977). Flight motor innervation of a flesh fly. Identified neurons and behavior of Arthropods.

[CR85] Jaslove SW, Brink PR, De Mello WC (1987). Electrotonic coupling in the nervous system. Cell-to-Cell communication.

[CR86] Johansson R, Westling G (1984). Roles of glabrous skin receptors and sensorimotor memory in automatic control of precision grip when lifting rougher or more slippery objects. Exp Brain Res.

[CR87] Johansson R, Westling G (1988). Coordinated isometric muscle commands adequately and erroneously programmed for the weight during lifting task with precision grip. Exp Brain Res.

[CR88] Josephson RK, Syme DA (2001). How to build fast muscles. II. asynchronous muscle: a design breakthrough. Am Zool.

[CR89] Josephson RK, Malamud JG, Stokes DR (2000). Asynchronous muscle: a primer. J Exp Biol.

[CR90] Kim AJ, Fitzgerald JK, Maimon G (2015). Cellular evidence for efference copy in *Drosophila* visuomotor processing. Nat Neurosci.

[CR91] Koenraadt KLM, Roelofsen EGJ, Duysens J, Keijsers N (2013). Cortical control of normal gait and precision stepping: an fNIRS study. Neuroimage.

[CR92] Kržič U, Vladimir R, Leonard KR, Linke WA, Bullard B (2010). Regulation of the oscillatory contraction in insect flight muscle by troponin. J Mol Biol.

[CR93] Lehmann F-O, Elsner N, Wässle H (1997). The changes of wing kinematics and power requirements during elevated force production in the genus *drosophila*. Neurobiology—from membrane to mind.

[CR94] Lehmann F-O (1999). Ambient temperature affects free-flight performance in the fruit fly *Drosophila melanogaster*. J Comp Physiol B.

[CR95] Lehmann F-O (2001). The efficiency of aerodynamic force production in *Drosophila*. Comp Biochem Physiol Part A.

[CR96] Lehmann F-O, Dickinson MH (1997). The changes in power requirements and muscle efficiency during elevated force production in the fruit fly, *Drosophila melanogaster*. J Exp Biol.

[CR97] Lehmann F-O, Götz KG (1996). Activation phase ensures kinematic efficacy in flight-steering muscles of *Drosophila melanogaster*. J Comp Physiol.

[CR98] Lehmann F-O, Pick S (2007). The aerodynamic benefit of wing–wing interaction depends on stroke trajectory in flapping insect wings. J Exp Biol.

[CR99] Lehmann F-O, Skandalis DA, Berthé R (2013). Calcium signalling indicates bilateral power balancing in the *Drosophila* flight muscle during manoeuvring flight. J R Soc Interface.

[CR100] Liu L, Wolf R, Ernst R, Heisenberg M (1999). Context generalization in *Drosophila* visual learning requires the mushroom bodies. Nature.

[CR101] Machin KE, Pringle JWS, Tamashige M (1962). The physiology of insect fibrillar muscle IV. The effect of temperature on a beetle flight muscle. Proc Roy Soc Lond B.

[CR102] Magwere T, Pamplona R, Miwa S, Martinez-Diaz P, Portero-Otin M, Brand MD, Partridge L (2006). Flight activity, mortality rates, and lipoxidative damage in *Drosophila*. J Geronto A.

[CR103] Maimon G, Straw AD, Dickinson MH (2010). Active flight increases the gain of visual motion processing in *Drosophila*. Nat Neurosci.

[CR104] Mischiati M, Lin H, Herold P, Imler E, Olberg R, Leonardo A (2014). Internal models direct dragonfly interception steering. Nature.

[CR105] Mokso R, Schwyn DA, Walker SM, Doube M, Wicklein M, Müller T, Stampanoni M, Taylor GK, Krapp HG (2015). Four-dimensional in vivo X-ray microscopy with projection-guided gating. Sci Rep.

[CR106] Moore JR, Vigoreaux JO (2006). Stretch activation: towards a molecular mechanism. Nature’s versatile engine: insect flight muscle inside and out.

[CR107] Mronz M, Lehmann F-O (2008). The free flight response of *Drosophila* to motion of the visual environment. J Exp Biol.

[CR108] Muijres FT, Elzinga MJ, Melis JM, Dickinson Michael H (2014). Flies evade looming targets by executing rapid visually directed banked turns. Science.

[CR109] Muijres FT, Elzinga MJ, Iwasaki NA, Dickinson Michael H (2015). Body saccades of *Drosophila* consist of stereotyped banked turns. J Exp Biol.

[CR110] Mureli S, Fox JL (2015). Haltere mechanosensory influence on tethered flight behavior in *Drosophila*. J Exp Biol.

[CR111] Nalbach G (1993). The halteres of the blowfly *Calliphora* I. kinematics and dynamics. J Comp Physiol A.

[CR112] Nalbach G (1994). Extremely non-orthogonal axes in a sense organ for rotation: behavioral analysis of the dipteran haltere system. Neuroscience.

[CR113] Nalbach G, Hengstenberg R (1994). The halteres of the blowfly *Calliphora* II. Three-dimensional organization of compensatory reactions to real and simulated rotations. J Comp Physiol A.

[CR114] Niven JE, Laughlin SB (2008). Energy limitation as a selective pressure on the evolution of sensory systems. J Exp Biol.

[CR115] Parsons MM, Krapp HG, Laughlin SB (2010). Sensor fusion in identified visual interneurons. Curr Biol.

[CR116] Peckham M, Cripps R, White D, Bullard B (1992). Mechanics and protein content of insect flight muscles. J Exp Biol.

[CR117] Pick S, Strauss R (2005). Goal-driven behavioral adaptations in gap-climbing *Drosophila*. Curr Biol.

[CR118] Pringle JWS (1948). The gyroscopic mechanism of the halteres of *Diptera*. Phil Trans R Soc Lond B.

[CR119] Pringle JWS (1968). Comparative physiology of the flight motor. Adv Insect Physiol.

[CR120] Pringle JWS (1978). Stretch activation of muscle: function and mechanism. Proc Roy Soc Lond B.

[CR121] Qiu F, Brendel S, Cunha PM, Astola N, Song B, Furlong EE, Leonard KR, Bullard B (2005). Myofilin, a protein in the thick filaments of insect muscle. J Cell Sci.

[CR122] Ramamurti R, Sandberg WC (2007). A computational investigation of the three-dimensional unsteady aerodynamics of *Drosophila* hovering and manoeuvring. J Exp Biol.

[CR123] Rheuben MB, Kammer AE (1987). Structure and innervation of the third axillary muscle of *Manduca* relative to its role in turning flight. J Exp Biol.

[CR124] Ristroph L, Bergou AJ, Berman GJ, Guckenheimer J, Wang ZJ, Cohen I, Childress S, Hosoi A, Schultz WW, Wang J (2012). Dynamics, control, and stabilization of turning flight in fruit flies. Natural locomotion in fluids and on surfaces.

[CR125] Rosner R, Egelhaaf M, Warzecha A-K (2010). Behavioural state affects motion-sensitive neurones in the fly visual system. J Exp Biol.

[CR126] Roth E, Sponberg S, Cowan NJ (2013). A comparative approach to closed-loop computation. Curr Opin Neurobiol.

[CR127] Samejima Y, Tsubaki Y (2010). Body temperature and body size affect flight performance in a damselfly. Behav Ecol Sociobiol.

[CR128] Sherman A, Dickinson MH (2003). A comparison of visual and haltere-mediated equilibrium reflexes in the fruit fly *Drosophila melanogaster*. J Exp Biol.

[CR129] Sherman A, Dickinson MH (2004). Summation of visual and mechanosensory feedback in *Drosophila* flight control. J Exp Biol.

[CR130] Shishkin A, Schützner P, Wagner C, Lehmann F-O, Tropea C, Bleckmann H (2012). Experimental quantification and numerical simulation of unsteady flow conditions during free flight maneuvers in insects. Nature-inspired fluid mechanics.

[CR131] Sotavalta O (1947). The flight-tone (wing stroke frequency) of insects. Acta Entomologica Fenn.

[CR132] Sponberg S, Daniel TL (2012). Abdication power for control: a precision timing strategy to modulate function of flight power muscles. Proc Roy Soc Lond B.

[CR133] Springthorpe D, Fernándes M, Hedrick T (2012). Neuromuscular control of free-flight turns in the hawkmoth *Manduca sexta*. J Exp Biol.

[CR134] Spüler M, Heide G (1978). Simultaneous recordings of torque, thrust and muscle spikes from the fly *Musca domestica* during optomotor responses. Z Naturforsch.

[CR135] Stafford N (2007). Spy in the sky. Nature.

[CR136] Stevenson RD, Josephson RK (1990). Effects of operating frequency and temperature on mechanical power output from moth flight muscle. J Exp Biol.

[CR137] Strausfeld NJ, Gronenberg W (1990). Descending neurons supplying the neck and flight motor of *Diptera*: organization and neuroanatomical relationships with visual pathways. J Comp Neurol.

[CR138] Swank DM (2012). Mechanical analysis of *Drosophila* indirect flight and jump muscles. Methods.

[CR139] Takei T, Seki K (2013). Spinal premotor interneurons mediate dynamic and static motor commands for precision grip in monkeys. J Neurosci.

[CR140] Tawada K, Kawai M (1990). Covalent cross-linking of single muscle fibers from rabbit psoas increases oscillatory power. Biophys J.

[CR141] Taylor GK, Krapp HG, Casas J, Simpson SJ (2007). Sensory systems and flight stability: what do insects measure and why?. Advances in insect physiology - Insect mechanics and control.

[CR142] Thelen DG, Anderson FC (2006). Using computed muscle control to generate forward dynamic simulations of human walking from experimental data. J Biomech.

[CR143] Thomas N, Thornhill RA (1995). Relaxation of tension in fibrillar insect flight muscle. J Physiol.

[CR144] Tohtong R, Yamashita H, Graham M, Haeberle J, Simcox A, Maughan D (1995). Impairment of muscle function caused by mutations of phosphorylation sites in myosin regulatory light chain. Nature.

[CR145] Trimarchi JR, Murphey RK (1997). The shaking-B^2^ mutation disrupts electrical synapses in a flight circuit in adult *Drosophila*. J Neurosci Methods.

[CR146] Trimarchi JR, Schneiderman AM (1994). The motor neurons innervating the direct flight muscles of *Drosophila melanogaster* are morphologically specialized. J Comp Neurol.

[CR147] Tu MS, Dickinson MH (1994). Modulation of negative work output from a steering muscle of the blowfly *Calliphora vicina*. J Exp Biol.

[CR148] Tu MS, Dickinson MH (1996). The control of wing kinematics by two steering muscles of the blowfly (*Calliphora vicina)*. J Comp Physiol A.

[CR149] Walker SM, Schwyn DA, Mokso R, Wicklein M, Müller T, Doube M, Stampanoni M, Krapp HG, Taylor GK (2014). In vivo time-resolved microtomography reveals the mechanics of the blowfly flight motor. PLoS Biol.

[CR150] Wang S, Li Y, Feng C, Guo A (2003). Dissociation of visual associative and motor learning in *Drosophila* at the flight simulator. Behav Process.

[CR151] Wang H, Ando N, Kanzaki R (2008). Active control of free flight manoeuvres in a hawkmoth, *Agrius convolvuli*. J Exp Biol.

[CR152] Wang Q, Zhao C, Swank DM (2011). Calcium and stretch activation modulate power generation in *Drosophila* flight muscle. Biophys J.

[CR153] Willigenburg NW, Kingma I, Hoozemans MJM, van Dieën JH (2013). Precision control of trunk movement in low back pain patients. Hum Mov Sci.

[CR154] Wilson DM (1961). The central nervous control of flight in a locust. J Exp Biol.

[CR155] Yan L-J, Sohal RS (2000). Prevention of flight activity prolongs the life span of the housefly, *Musca domestica*, and attenuates the age-associated oxidative damage to specific mitochondrial proteins. Free Radic Biol Med.

[CR156] Yang JF, Gorassini M (2006). Spinal and brain control of human walking: implications for retraining of walking. Neuroscientist.

[CR157] Yoda K, Kohno H, Naito Y (2004). Development of flight performance in the brown booby. Proc Roy Soc Lond B.

[CR158] Zhao Y, Kawai M (1993). The effect of lattice spacing change on cross-bridge kinetics in chemically skinned rabbit psoas muscle fibers: II. Elementary steps affected by the spacing change. Biophys J.

[CR159] Zucker RS (1989). Short-term synaptic plasticity. Annu Rev Neurosci.

